# Outcomes and factors associated with prolonged stays among patients admitted to adult intensive care unit in a resource-limited setting: a multicenter chart review

**DOI:** 10.1038/s41598-024-64911-x

**Published:** 2024-06-17

**Authors:** Tola Getachew Bekele, Birhanu Melaku, Lemlem Beza Demisse, Legese Fekede Abza, Awol Seid Assen

**Affiliations:** 1 Department of Nursing, Jimma University Medical Center, Jimma, Ethiopia; 2 Department of Emergency Medicine, College of Health Science, Addis Ababa University, Addis Ababa, Ethiopia; 3 Department of Nursing, College of Medicine and Health Sciences, Wolkite University, Wolkite, Ethiopia

**Keywords:** Intensive care unit, Prolonged stay, Outcome, Length of stay, Addis Ababa, Ethiopia, Health care, Medical research

## Abstract

The length of stay in an intensive care unit is used as a benchmark for measuring resource consumption and quality of care and predicts a higher risk of readmission. The study aimed to assess the outcome and factors associated with prolonged intensive care unit stays among those admitted to adult intensive care units of selected public hospitals in Addis Ababa from January 1, 2022, to December 31, 2022. A multicenter retrospective chart review was conducted involving 409 adult patients. Binary logistic regression was used to assess factors associated with a prolonged stay and chi-square tests were used to assess associations and differences in outcomes for prolonged stays. The study, involving 409 of 421 individuals, revealed a predominantly male (55.0%) and the median age of study participants was 38, with an interquartile range (27, 55). Approximately 16.9% experienced prolonged stays, resulting in a 43.5% mortality rate. After adjustments for confounders, there were significant associations with prolonged stays for sedative/hypnotics, readmission, and complications. The study revealed that for every six patients admitted to the intensive care unit, one patient stayed longer, with nearly half experiencing mortality, demanding increased attention. The study emphasized the critical need for improvement in addressing associations between sedative/hypnotics, readmissions, complications, and prolonged stays.

## Introduction

The intensive care unit (ICU) is a structured system for treating seriously ill patients and consists of all-inclusive and skilled nursing and medical care, increased monitoring capability, and a variety of physiologic organ support to keep patients alive during a period of life-threatening organ system failure^1^. The provision of sophisticated interventions for critically ill patients has made the ICU one of the most resource and time-consuming departments in the medical sector^2,3^.

The length of stay is the primary indicator used by the World Health Organization (WHO) report to monitor and evaluate the performance of hospitals^4^. The length of stay in the ICU is a significant factor in determining hospital costs, an indicator of treatment quality^5^. In addition, it is used as a benchmark for measuring resource consumption and predicts a higher risk of readmission^5,6^. Most ICU patients stay for only a few days^7,8^. Nevertheless, only a small percentage of patients who had a serious disease that was persistent or chronic stayed for more than a week, which is a prolonged stay^7^.

Different authors define a prolonged stay as anything lasting longer than 3 to 30 days, while other authors define the term by looking at the distribution of ICU stays and identifying a threshold tail that indicates a prolonged stay^3,8–14^. Despite differences in the definition of a prolonged stay in the ICU, studies have repeatedly shown a small percentage (7% to 11%) of patients with a prolonged ICU stay^13^. In a study conducted in developing countries such as Nigeria, the incidence of prolonged stays greater than 14 days in the ICU was 40.34%^15^. In Ethiopia patients had unusually high durations of prolonged ICU stays despite employing different cutoff points. According to research conducted at Addis Ababa, almost 50% of patients stayed in the ICU more than 7 days^16^, and at the Nigist Eleni Mohammed Memorial Hospital, approximately 52.9% of patients stayed there longer than 3 days^14^.

Despite differing cutoff points for prolonged stays, prior research from various countries showed that the mortality rate for individuals requiring a prolonged stay ranged from 21.1 to 70.58%^3,7,11,12,17,18^. A diagnosis at admission, advanced age, male sex, comorbidity, postoperative sepsis or septic shock, preoperative illnesses^5^, pulmonary diseases^10^, a low GCS^19^, and a requirement for organ support^20,21^ were the factors that contributed to a prolonged ICU stay. There is limited literature on the factors that affect prolonged stays in the ICU in Ethiopia. Most studies conducted in ICUs focus on patient outcomes and rely on length of stay as an indicator of mortality. The current study aims to fill this knowledge gap by identifying factors associated with prolonged ICU stays that can help improve patient care and outcomes in ICUs.

## Methods and materials

### Study design and setting

An institution-based retrospective chart review was conducted in Addis Ababa from March 28–April 28, 2023. Addis Ababa, the capital and largest city of Ethiopia, with a population of 5,005,524 and a land area of 527 km^2^, serves as the headquarters for both the African Union and the Economic Commission for Africa. The city has 11 subcities, 116 woredas, 40 private hospitals, 98 health facilities, and 13 public hospitals. The Addis Ababa Health Bureau administers six of the 13 public hospitals; the Federal Ministry of Health administers six; and Addis Ababa University administers one. Amanuel Mental Health Hospital was excluded because of a lack of ICU care^16,22,23^. The research was carried out at four public hospitals in Addis Ababa, which were selected by simple random sampling. These are Tikur Anbessa Specialized Hospital, Zewditu Memorial Hospital, Menelik II Referral Hospital, and Yekatit 12 Hospital Medical College.

### Source and study population

The source of the population was all patients admitted and treated at the adult ICUs of selected public hospitals in Addis Ababa from January 1, 2022, to December 31, 2022.

The study population sampled patients who were admitted and treated at the adult ICUs of selected public hospitals in Addis Ababa from January 1, 2022, to December 31, 2022.

### Inclusion and exclusion criteria

All patients who were admitted to the ICU in four selected public hospitals in Addis Ababa from January 1, 2022, to December 31, 2022, and who had a complete medical chart from the day of admission until discharge, were included in the study.

### Sample size determination

The total sample size required for this study was calculated based on the outcome of interest.

### For the first and second specific objectives

The sample size was calculated using a single proportion population formula. For first specific objective, based on a study conducted at Nigist Eleni Mohammed Memorial Hospital, Hosanna^14^ reported that the magnitude of prolonged stay in the ICU was 52.9%, and for second specific objective, according to a study conducted at a tertiary hospital in Nigeria^24^ the mortality rate of patients who had a prolonged stay in the ICU was 37.8%, with a 95% level of confidence, and a 5% margin of error, which gives sample size of 383 and 361 respectively.

The sample size was estimated using the following assumptions:

$$n=\frac{{\left(Z\frac{\alpha }{2}\right)}^{2}P\left(1-P\right)}{{d}^{2}}$$

where, n = minimum sample size, Zα/2 = Z value at (α = 0.05) = 1.96, d = margin of error (0.05), p = 0.529 for first specific objective, 0.378 for second specific objective.

### For the third specific objective:

The sample size was determined using the double population proportion. Sex was associated with a longer stay in an intensive care unit, according to a study conducted at Nigist Elleni Mohammed Memorial Hospital in Hosanna^14^.

From this, the outcome of the exposed group (male) was 63.19%, the outcome of the unexposed group (female) was 38.46%, the ratio of the unexposed group to the exposed group was 1, the confidence interval was 95%, and the power was 80%. The sample size was 142.

Of those three calculated sample sizes, the largest sample size was 383.

By adding a nonresponse rate (chart loss rate) of 10%, the final sample size was 421.

### Sampling procedure

**Figure 1:**
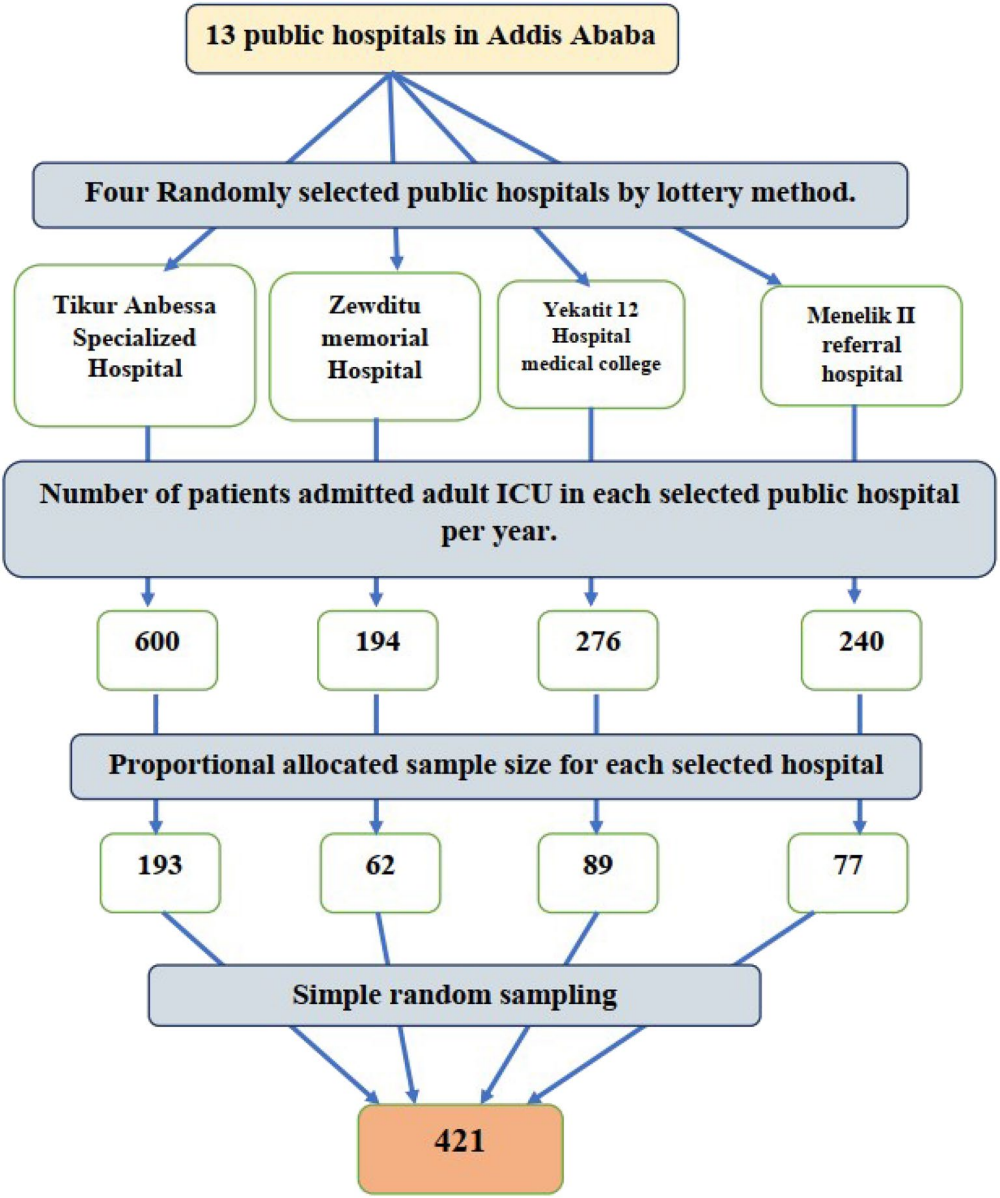
Shows sampling procedure.

Four public hospitals (Tikur Anbessa Specialized Hospital, Zewditu Memorial Hospital, Menelik II Referral Hospital, and Yekatit 12 Hospital Medical College) were selected by simple random sampling from 12 public hospitals. Then, to select the patient charts from each hospital, the proportional allocation formula was used based on the number of patients admitted to the ICU. After that, medical records of patients admitted to the adult ICU were identified from the logbook in each hospital, and a simple random sampling technique was applied to select the patient charts (Fig. 1).

### Variables

#### Dependent variable

Primary outcome: Prolonged ICU stay.

Secondary outcome: Outcome of prolonged ICU stays.

#### Independent variables

- Sociodemographic variables (age, sex, residence)
- Clinical characteristics of the patient (comorbidity, diagnosis at admission, source of admission)
- Vital signs at admission (systolic blood pressure, pulse rate, breathing rate, oxygen saturation) and Glasgow Coma Scale score at admission.
- Laboratory investigation at admission (sodium, potassium, chloride, complete blood count, serum creatinine, blood urea nitrogen)
- Intervention given during ICU stay (mechanical ventilation, inotropes/vasopressors, tracheostomy)
- Types of surgery (emergency vs elective)
- Complications developed while staying in the ICU (acute kidney injury, bedsore, ventilator associated pneumonia, hospital acquired infection).

### Operational definition

*Prolonged ICU stay:* according to this study those who stayed in the ICU for more than 14 days^6,10,18,21,25,26^.

*Outcome:* In this study outcome was interpreted as the status of patients after ICU admission, whether they survived or died during discharge.

### Data collection tools and procedures

A pretested structured data retrieval form was developed from different related literatures^5,8,10,18–20,27–31^. It has five sections: sociodemographic characteristics, clinical characteristics, vital signs and laboratory investigations at admission, treatment given during the ICU stay, and patient outcomes. The data were collected by four BSc nurses and two MSc nurse supervisors. The data were gathered by reviewing patient medical records via the Kobo Collect v2022.4.4 Android application. The collected data were stored in Excel on the Kobo Toolbox cloud under the principal investigator's account.

### Data quality assurance

To prevent redundancy, a unique tag was placed on all the charts. Prior to the actual data collection period, a pretest was administered to 5% of the study sample at Tikur Anbessa Specialized Hospital 1 week before actual data collection to make any necessary changes based on the results, although no changes were considered necessary. The principal investigator provided training to the data collectors, providing an overview of the study's objectives and the entire step-by-step data collection process, including instructions on how to use Kobo to collect data using an Android application. On-site supervision was given to resolve any uncertainty about the data collection instruments and methods. At the end of each data collection day, completed checklists were checked on the cloud of the Kobo Toolbox to ensure completeness and consistency.

### Data processing and analysis

The collected data were downloaded from the cloud of KoboToolbox in XLS and SPSS labels and exported to the Statistical Package for the Social Sciences (SPSS) version 27 for editing, coding, and further analysis.

For categorical data, descriptive data were presented as frequencies and percentages. For continuous variables, the means, medians, interquartile ranges, and standard deviations were presented as appropriate. Binary logistic regression was used to assess factors associated with a prolonged stay in the ICU. Variables with p < 0.25 in the bivariable analysis were included in the multivariable logistic regression analysis. An adjusted odds ratio (AOR) with a corresponding 95% confidence interval (CI) was used to show the strength of the association between prolonged ICU stay and independent variables at a P-value ≤ 0.05 as the cutoff point. The Hosmer and Lemeshow test showed good model fit. A chi-square test or Fisher's exact test was used to determine whether there was a significant difference between the outcome of a prolonged stay in the ICU and the independent variables. Variables with P values less than 0.05 were significantly different.

### Ethics approval

Verbal or written consent was not obtained from the study participants because the data were collected from the patient’s medical charts and there was no direct contact between the patients and the data collectors. Addis Ababa University College of Health Science, Department of Emergency Medicine (Reference No.: EM/SM/877/2015), and Addis Ababa City Administration Health Bureau ethical committee (Reference No.: A/A/11193/227) approved the informed consent waiver. The Addis Ababa City Administration Health Bureau ethical committee chairperson, Dr. Yohannis W/Kidan, secretary, Mr. Tesfaye Desalign, and member, Mr. Biniam Abebe (email addresses: yoha2wok@yahoo.com, tesfayedes62@gmail.com, abe_bini@yahoo.com), respectively. The experimental protocol was approved by the Addis Ababa University College of Health Science, Department of Emergency Medicine, the department head, and chairperson was Dr. Temesgen Beyene (Yetemeaau2001@gmail.com).

The letter of permission was written to the hospitals, and to the hospital managers, research officers, and the health management information system focal persons who verbally consented to the use of the patient's medical charts for data collection. The study was conducted following the relevant guidelines, regulations, and principles of the Helsinki Declaration. All the data obtained during the study were kept confidential, only accessible by the research team, and used solely for the purpose of the research. For data collection purposes, medical record numbers were utilized, and no personal identifiers were gathered or utilized in the research.

## Results

### Sociodemographic and clinical characteristics

A total of 409 of the 421 patient charts included in the study were studied.. Over half of the participants (55.0%) were male, and (58.2%) were from Addis Ababa. The median age was 38 years, with an interquartile range of 27 to 55 years and approximately 36.4% of participants were older than 45 years. Most participants were admitted from emergency departments (45.7%), followed by 37.7% from the operating room (OR). Among those admitted from the OR, the majority (72.7%) underwent emergency surgery. New admissions accounted for 95.8% of the total frequency of admissions. Approximately 39.4% of participants had comorbidities, with hypertension being the most prevalent (48.1%), followed by heart diseases (21.9%), and diabetes mellitus (21.3%) (Table 1).

**Table 1:** Socio-demographic and clinical characteristics of patients admitted to adult intensive care units at selected public hospitals in Addis Ababa from January 1, 2022, to December 31, 2022 (n = 409).

Variables	Categories	ICU length of stay		Total
		< 14 days	≥ 14 days	
Gender	Male	181 (44.3%)	44 (10.8%)	225 (55.0%)
	Female	159 (38.9%)	25 (6.1%)	184 (45.0%)
Age (years)	18–30	122 (29.8%)	23 (5.6%)	145 (35.5%)
	31–45	97 (23.7%)	18 (4.4%)	115 (28.1%)
	> 45	121 (29.6%)	28 (6.8%)	149 (36.4%)
Residence	Addis Ababa	193 (47.2%)	45 (11.0%)	238 (58.2%)
	Out of Addis Ababa	147 (35.9%)	24 (5.9%)	171 (41.8%)
Source of admission	Emergency department	146 (35.7%)	41 (10.0%)	187 (45.7%)
	In hospital wards	56 (13.7%)	12 (2.9%)	68 (16.6%)
	Operation room	138 (33.7%)	16 (3.9%)	154 (37.7%)
If source of admission from OR type of surgery	Emergency	105 (68.2%)	7 (4.5%)	112 (72.7%)
	Elective	33 (21.4%)	9 (5.8%)	42 (27.3%)
Cause surgery due to trauma	Yes	5 (3.2%)	5 (3.2%)	10 (6.5%)
	No	133 (86.4%)	11 (7.1%)	144 (93.5%)
Frequency of ICU admission	New admission	332 (81.2%)	60 (14.7%)	392 (95.8%)
	Readmission	8 (2.0%)	9 (2.2%)	17 (4.2%)
Comorbidity	Yes	131 (32.0%)	30 (7.3%)	161 (39.4%)
	No	209 (51.1%)	39 (9.5%)	248 (60.6%)
Types of comorbidities	Hypertension	63 (39.4%)	14 (8.8%)	77 (48.1%)
	Diabetic mellitus	28 (17.5%)	6 (3.8%)	34 (21.3%)
	Heart diseases	31 (19.4%)	4 (2.5%)	35 (21.9%)
	HIV	21 (13.1%)	6 (3.8%)	27 (16.9%)
	Chronic lung diseases	13 (8.1%)	2 (1.3%)	15 (9.4%)
	Others^a^	38 (23.8%)	10 (6.3%)	48 (30.0%)

^a^Arthritis, chronic liver disease, dyslipidemia, epilepsy, hyperthyroidism, idiopathic thrombocytopenic purpura, myasthenia gravis, peripheral arterial disease, renal disease, systemic lupus erythematous, ulcerative colitis.

**Figure 2:**
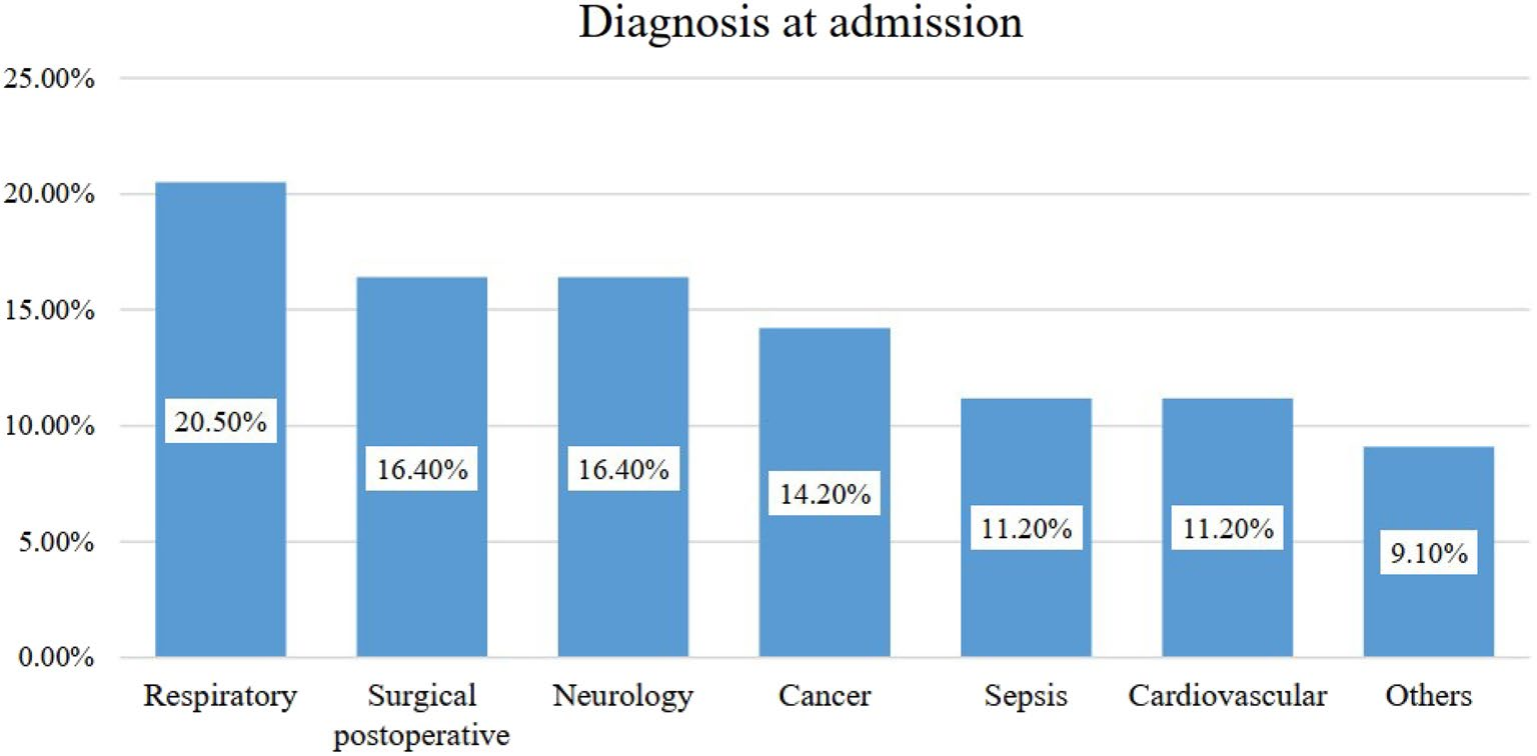
Diagnosis at admission of patients admitted to adult intensive care units at selected public hospitals in Addis Ababa from January 1, 2022, to December 31, 2022 (n = 409).

In terms of diagnosis at admission, respiratory conditions were the most common 84 (20.5%), followed by surgical postoperative conditions and neurological conditions 67 (16.4%) patients each (Fig. 2).

### Prevalence of prolonged stays

**Figure 3:**
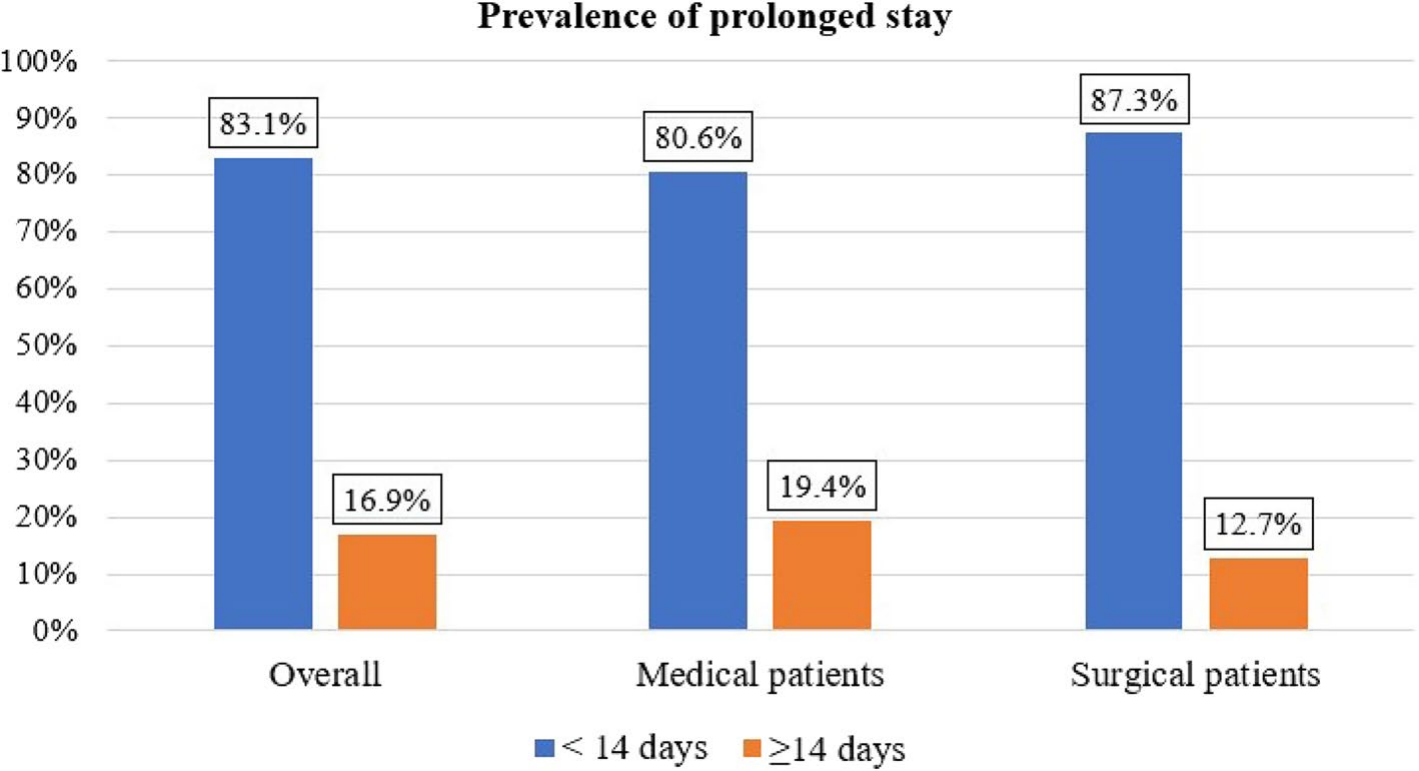
Prevalence of prolonged ICU stay among those admitted to adult intensive care units at selected public hospitals in Addis Ababa from January 1, 2022, to December 31, 2022.

The overall prolonged stay of study participants was 69 (16.9%, (95% CI = 13.5–20.5%)). Approximately 49 (19.4%) medical patients stayed longer, but approximately 20 (12.7%) surgical patients stayed longer (Fig. 3).

### Vital signs and laboratory investigation at admission

According to the complete blood analysis of the study participants, approximately 178 (43.5%) had anemia, whereas only 11 (2.7%) had polycythemia. More than half of the participants had abnormal white blood cell counts, 30 7.4%) had leukopenia and 188 (46.1%) had leukocytosis. Nearly two thirds (64.8%) of the participants had a normal platelet count.

More than one third of the participants were observed to have abnormal serum sodium levels, with approximately 29.6% having hyponatremia and only 9.5% having hypernatremia. More than two-fifths of the participants, 179 (43.7%), had deranged potassium levels. Nearly one-fourth of the participants (24.4%) had hypokalemia, while almost one-fifth (19.3%) had hyperkalemia. The study participants' liver function tests indicated that elevated AST, ALT, and ALP levels were present in 37.6%, 27.0%, and 5.1% of the patients, respectively. Additionally, renal function tests revealed that 20.8% of the patients had increased blood urea nitrogen (BUN) values and 25.2% had elevated creatinine levels.

The vital signs at admission, including deranged vital signs seen at admission (tachycardia, tachypnea, and hypotension), were recorded in 189 (46.2%), 162 (39.6%), and 48 (11.7%) patients, respectively. The pupillary response at admission was reactive in the majority of the study participants (366; 89.5%). More than two-thirds of participants had a GCS score ranging from 13 to 15, followed by a GCS ranging from 9 to 12, 94 (23.0%), and a GCS less than 8, 53 (13.0%) (Table 2).

**Table 2:** Vital signs and laboratory findings of patients admitted to adult intensive care units at selected public hospitals in Addis Ababa from January 1, 2022, to December 31, 2022 (n = 409).

Variables	Categories	ICU length of stay		Total
		< 14 days	≥ 14 days	
White blood count (cells/L)	4.5–11	160 (39.2%)	30 (7.4%)	190 (46.6%)
	< 4.5	29 (7.1%)	1 (0.2%)	30 (7.4%)
	> 11	151 (37.0%)	37 (9.1%)	188 (46.1%)
Hemoglobin (mg/dl)	12–18	182 (44.5%)	38 (9.3%)	220 (53.8%)
	< 12	149 (36.4%)	29 (7.1%)	178 (43.5%)
	> 18	9 (2.2%)	2 (0.5%)	11 (2.7%)
Sodium (mEq/l)	135–145	204 (49.9%)	45 (11.0%)	249 (60.9%)
	< 135	105 (25.7%)	16 (3.9%)	121 (29.6%)
	> 145	31 (7.6%)	8 (2.0%)	39 (9.5%)
Potassium (mEq/l)	3.5–4.5	192 (46.9%)	38 (9.3%)	230 (56.2%)
	< 3.5	80 (19.6%)	20 (4.9%)	100 (24.4%)
	> 4.5	68 (16.6%)	11 (2.7%)	79 (19.3%)
Aspartate transaminase (IU/L)	6–40	217 (53.3%)	37 (9.1%)	254 (62.4%)
	> 40	121 (29.7%)	32 (7.9%)	153 (37.6%)
Alanine transaminase (IU/L)	6–40	245 (60.8%)	49 (12.2%)	294 (73.0%)
	> 40	89 (22.1%)	20 (5.0%)	109 (27.0%)
Alkaline phosphatase (IU/L)	0–270	323 (79.0%)	65 (15.9%)	388 (94.9%)
	> 270	17 (4.2%)	4 (1.0%)	21 (5.1%)
Blood Urea Nitrogen	≤ 40	266 (65.0%)	58 (14.2%)	324 (79.2%)
	> 40	74 (18.1%)	11 (2.7%)	85 (20.8%)
Serum creatinine (mg/dl)	≤ 1.2	249 (60.9%)	57 (13.9%)	306 (74.8%)
	> 1.2	91 (22.2%)	12 (2.9%)	103 (25.2%)
Pulse rate at admission (beats/min)	60–100	190 (46.5%)	30 (7.3%)	220 (53.8%)
	> 100	150 (36.7%)	39 (9.5%)	189 (46.2%)
Systolic blood pressure at admission (mmHg)	≥ 90	297 (72.6%)	64 (15.6%)	361 (88.3%)
	< 90	43 (10.5%)	5 (1.2%)	48 (11.7%)
Respiratory rate at admission (Breaths/min)	16–24	213 (52.1%)	34 (8.3%)	247 (60.4%)
	> 24	127 (31.1%)	35 (8.6%)	162 (39.6%)
Mean arterial pressure (mmHg)	70–100	226 (55.3%)	47 (11.5%)	273 (66.7%)
	< 70	51 (12.5%)	7 (1.7%)	58 (14.2%)
	> 100	63 (15.4%)	15 (3.7%)	78 (19.1%)
Oxygen saturation at admission (%)	≥ 90	305 (74.6%)	61 (14.9%)	366 (89.5%)
	< 90	35 (8.6%)	8 (2.0%)	43 (10.5%)
Pupillary response at admission	Reactive	309 (75.6%)	57 (13.9%)	366 (89.5%)
	Non-Reactive	31 (7.6%)	12 (2.9%)	43 (10.5%)
Glasgow Coma Scale at admission	13–15	233 (57.0%)	29 (7.1%)	262 (64.1%)
	9–12	73 (17.8%)	21 (5.1%)	94 (23.0%)
	< 8	34 (8.3%)	19 (4.6%)	53 (13.0%)

### Treatment given and complications developed during the ICU stay

Regarding treatment given during the ICU stay: H2 blockers/ proton pump inhibitors were given to 335 patients (81.9%), thrombolytics/anticoagulants to 229 patients (56%), sedatives/hypnotics to 94 patients (23.0%), vasopressors and inotropes to 81 patients (19.8%), blood product transfusion to 71 patients (17.4%), mechanical ventilation to 182 patients (44.5%), and tracheostomy to 34 patients (8.3%). Among those patients who received mechanical ventilation, the majority, 176 (96.7%), received invasive ventilation, and more than half, 106 (58.6%), of those patients were on mechanical ventilation for less than 7 days. During their ICU stay, 107 patients (26.2%) experienced complications. The most common complications were hospital-acquired infections in 37 patients (35.6%), followed by acute kidney injury in 31 patients (29.8%), and pressure sores in 30 patients (28.8%) (Table 3).

**Table 3:** Treatments given and complications that developed during the stays of patients admitted to adult intensive care units at selected public hospitals in Addis Ababa from January 1, 2022, to December 31, 2022 (n = 409).

Variables	Categories	ICU Length of stay		Total
		< 14 days	≥ 14 days	
H2 blockers/proton pump inhibitors	Yes	269 (65.8%)	66 (16.1%)	335 (81.9%)
	No	71 (17.4%)	3 (0.7%)	74 (18.1%)
Thrombolytics/anticoagulants	Yes	62 (15.2%)	167 (40.8%)	229 (56.0%)
	No	7 (1.7%)	173 (42.3%)	180 (44.0%)
Sedatives/hypnotics	Yes	59 (14.4%)	35 (8.6%)	94 (23.0%)
	No	281 (68.7%)	34 (8.3%)	315 (77.0%)
Vasopressors/inotropes	Yes	70 (17.1%)	11 (2.7%)	81 (19.8%)
	No	270 (66.0%)	58 (14.2%)	328 (80.2%)
Infusion dose of vasopressors/inotropes (mcg/kg/min)	Minimal support (0.01–0.05)	8 (9.9%)	2 (2.5%)	10 (12.3%)
	Moderate support (0.05–0.5)	37 (45.7%)	4 (4.9%)	41 (50.6%)
	Maximal support (> 0.5)	25 (30.9%)	5 (6.2%)	30 (37.0%)
Blood product transfusion	Yes	56 (13.7%)	15 (3.7%)	71 (17.4%)
	No	284 (69.4%)	54 (13.2%)	338 (82.6%)
Types of blood product	Whole blood	8 (11.3%)	6 (8.5%)	14 (19.7%)
	Packed red blood cell	41 (57.7%)	12 (16.9%)	53 (74.6%)
	Platelet	41 (57.7%)	12 (16.9%)	53 (74.6%)
	Fresh frozen plasma	14 (19.7%)	3 (4.2%)	17 (23.9%)
Mechanical ventilation	Yes	125 (30.6%)	57 (13.9%)	182 (44.5%)
	No	215 (52.6%)	12 (2.9%)	227 (55.5%)
Types mechanical ventilation	Invasive	120 (65.9%)	56 (30.8%)	176 (96.7%)
	Noninvasive	5 (2.7%)	1 (0.5%)	6 (3.3%)
Length of stay on mechanical ventilation (days)	< 7	100 (55.2%)	6 (3.3%)	106 (58.6%)
	7–14	22 (12.2%)	14 (7.7%)	36 (19.9%)
	15–21	0 (0.0%)	12 (6.6%)	12 (6.6%)
	> 21	3 (1.7%)	24 (13.3%)	27 (14.9%)
Tracheostomy	Yes	11 (2.7%)	23 (5.6%)	34 (8.3%)
	No	329 (80.4%)	46 (11.2%)	375 (91.7%)
Complication	Yes	56 (13.7%)	51 (12.5%)	107 (26.2%)
	No	284 (69.4%)	18 (4.4%)	302 (73.8%)
Types complication	Hospital acquired infections	17 (16.3%)	20 (19.2%)	37 (35.6%)
	Acute kidney injury	19 (18.3%)	12 (11.5%)	31 (29.8%)
	Pressure sore	6 (5.8%)	24 (23.1%)	30 (28.8%)
	Electrolyte abnormality	18 (17.3%)	8 (7.7%)	26 (25.0%)
	Ventilator associated pneumonia	3 (2.9%)	15 (14.4%)	18 (17.3%)
	Hypoalbuminemia	6 (5.8%)	12 (11.5%)	18 (17.3%)
	Upper gastrointestinal bleeding	10 (9.6%)	6 (5.8%)	16 (15.4%)
	Others^a^	12 (11.5%)	17 (16.3%)	29 (27.9%)

^a^Anemia, catheter-associated urinary infection, deep vein thrombosis, drug induced liver injury, pulmonary embolism, tension pneumothorax, thrombocytopenia, thrombophlebitis.

### Patient outcomes

The overall mortality rate was 36.9%. Among 69 longer stays patients (≥ 14 days) in the ICU 30 of them died which accounts 43.5% (95% CI = 31.9%-55.1%). In comparison with shorter stays (< 14 days), out of 340 patients 121 of them died which accounts 35.6%, while nearly two-thirds of patients with shorter stays survived and most survivors 243 (94.2%) were transferred to irrespective wards (Table 4).

**Table 4:** Outcomes of patients admitted to adult intensive care units at selected public hospitals in Addis Ababa from January 1, 2022, to December 31, 2022 (n = 409).

Variables	Categories	ICU Length of stay		Total
		< 14 days	≥ 14 days	
Outcome	Survived	219 (64.4%)	39 (56.5%)	258 (63.1%)
	Died	121 (35.6%)	30 (43.5%)	151 (36.9%)
Survived	Transferred to irrespective wards	205 (93.6%)	38 (97.4%)	243 (94.2%)
	Discharged to home	8 (3.7%)	0 (0.0%)	8 (3.1%)
	Referred to other health facility	2 (0.9%)	1 (2.6%)	3 (1.2%)
	Self-discharged against medical advice	4 (1.8%)	0 (0.0%)	4 (1.6%)

### Factors associated with a prolonged ICU stay

Sex, source of admission, frequency of admission, diagnosis at admission, SBP at admission, RR at admission, PR at admission, AST, serum creatinine, sedatives/hypnotics, mechanical ventilation, and complications were found to be associated with prolonged ICU stay in a binary logistic regression analysis at a P-value less than 0.25. After adjusting for potential confounders, sedatives/hypnotics, frequency of admission, and complications were found to be significantly associated with prolonged ICU stays at a P-value less than 0.05 in a multivariable binary logistic regression.

The odds of staying longer among those readmitted to the ICU were 3.6 times higher than those who were first admitted (AOR = 3.6, 95% CI = 1.06–12.58, P value = 0.04). The odds of staying longer in the ICU among those who took sedative agents were 3.35 times higher compared to those who did not take them (AOR = 2.75, 95% CI = 1.26–6.01, P-value = 0.011). Regarding complications, the odds of staying longer among patients who developed complications were eight times higher compared to those who did not develop complications (AOR = 8.39, 95% CI = 4.14–16.9, P value = 0.0001) (Table 5).

**Table 5:** Distribution of factors associated with prolonged intensive care unit stays among those admitted to adult intensive care units at selected public hospitals in Addis Ababa from January 1, 2022, to December 31, 2022 (n = 409).

Variables	Categories	ICU Length of stay		COR 95% CI	AOR 95% CI	P-value
		≥ 14 days (n = 69)	< 14 days (n = 340)			
Gender	Male	44 (10.8%)	181 (44.3%)	1.5 (0.9, 2.6)	1.5 (0.75, 2.99)	0.244
	Female	25 (6.1%)	159 (38.9%)	1	1	
Source of admission	Emergency department	41 (10.0%)	146 (35.7%)	2.4 (1.3, 4.5)	3.8 (0.65, 22.4)	0.138
	In hospital wards	12 (2.9%)	56 (13.7%)	1.85 (0.8, 4.1)	2.65 (0.36,19.12)	0.33
	OR	16 (3.9%)	138 (33.7%)	1	1	
Frequency of ICU admission	New admission	60 (14.7%)	332 (81.2%)	1	1	
	Readmission	9 (2.2%)	8 (2.0%)	6.2 (2.3, 16.7)	3.6 (1.06, 12.58)	0.04*
Pulse rate (beats/min)	60–100	30 (7.3%)	190 (46.5%)	1	1	
	> 100	39 (9.5%)	150 (36.7%)	1.6 (0.97, 2.7)	1.6 (0.81, 3.3)	0.167
Systolic blood pressure (mmHg)	≥ 90	64 (15.6%)	297 (72.6%)	1	1	
	< 90	5 (1.2%)	43 (10.5%)	1.8 (0.69, 4.7)	0.59 (0.19, 1.8)	0.38
Respiratory rate (breaths/min)	16–24	34 (8.3%)	213 (52.1%)	1	1	
	> 24	35 (8.6%)	127 (31.1%)	1.7 (1.02, 2.9)	1.1 (0.53, 2.3)	0.77
Aspartate transaminase (IU/L)	6–40	37 (9.1%)	217 (53.3%)	1	1	
	> 40	32 (7.9%)	121 (29.7%)	1.5 (0.9, 2.6)	1.26 (0.63, 2.54)	0.507
Serum creatinine (mg/dl)	≤ 1.2	57 (13.9%)	249 (60.9%)	1	1	
	> 1.2	12 (2.9%)	91 (22.2%)	0.57 (0.29, 1.12)	0.44 (0.18, 1.05)	0.064
Sedatives	Yes	35 (8.6%)	59 (14.4%)	4.9 (2.8, 8.49)	2.75 (1.26, 6.01)	0.011*
	No	34 (8.3%)	281 (68.7%)	1	1	
Mechanical ventilation	Yes	57 (13.9%)	125 (30.6%)	8.1 (4.2, 15.8)	2.4 (0.99, 5.8)	0.052
	No	12 (2.9%)	215 (52.6%)	1	1	
Complication	Yes	51 (12.5%)	56 (13.7%)	14.36 (7.8, 26.4)	8.39 (4.14, 16.9)	0.0001*
	No	18 (4.4%)	284 (69.4%)	1	1	

Significant values are in bold.

**Statistically significant p-value < 0.05; 1: Reference.

### Comparison of the outcomes of prolonged ICU stays

According to the results of the chi-square test/Fisher's exact test, there was a significant difference in the outcome of patients who had prolonged ICU stays across groups categorized by the presence of complications such as acute kidney injury, pressure sores, and upper gastrointestinal bleeding, as well as among those who received vasopressors. The findings indicated that a significantly higher proportion of participants with acute kidney injury, pressure sores, and upper gastrointestinal bleeding died compared to those without such diseases. Additionally, a significantly higher proportion of participants who received vasopressors also died compared to those who did not receive vasopressors (Table 6).

**Table 6:** Comparison of the outcomes of patients with prolonged ICU stays.

Variables	Categories	Outcome of prolonged ICU stay		P-value
		Died (N%) n = 30	Survived (N%) n = 39	
Vasopressors	Yes	8 (72.7)	3 (27.3)	**0.033**
	No	22 (37.9)	36 (62.1)	
Complication	Yes	28 (54.9)	23 (45.1)	**0.001**
	No	2 (11.1)	16 (88.9)	
Acute kidney injury	Yes	11 (91.7)	1 (8.3)	** < 0.001**
	No	19 (33.3)	38 (66.7)	
Pressure sore	Yes	15 (62.5)	9 (37.5)	**0.020**
	No	15 (33.3)	30 (66.7)	
Upper gastrointestinal bleeding	Yes	5 (83.3)	1 (16.7)	**0.039**
	No	25 (39.7)	38 (60.3)	

Significant values are in bold.

## Discussion

Determining the length of stay in the ICU is a key component of the healthcare system. As a result, study findings on the prevalence, risk factors, and mortality of patients with a prolonged ICU stay were crucial for effective resource allocation, dealing with high-risk groups for prolonged stays, and evaluating various treatment modalities and interventions to reduce the length of stay in the ICU and improve patient outcomes. This retrospective chart review study was designed to assess the outcomes and factors related to prolonged stays in 409 patients admitted to adult intensive care units in selected public hospitals in Addis Ababa between January 1, 2022, and December 31, 2022.

According to the present study, the prevalence of prolonged ICU stays was 16.9% (95% CI =  13.5–20.5%), which indicated that one patient stayed longer than 14 days for every six patients admitted to the ICU. This finding aligns with several studies; for example, a study conducted in Barbados^25^, Taiwan^31^, Turkey^10,20^, and Thailand^32^ ranged from 13.2 to 20.1%.

This finding was high compared to those of studies conducted in America^30^, Brazil^21^, Switzerland^7^, France^8^, Taiwan^26^, Turkey^3,12^, Saudi Arabia^6^, and Nigeria^24^ which reported values ranging from 5 to 11.3%. This difference was due to the use of different cutoff points to define prolonged stays; in this study, the cutoff point was 14 days, but studies conducted in France and Turkey had cutoff points of greater than 30 days and 28 days, respectively. The other attributing factors for this difference were differences in the quality and capacity of the healthcare system, availability and accessibility of medical infrastructure, availability of rehabilitation services, and sample size.

This result was low when compared to that of a study conducted in southern Ethiopia^14^ in which 52.9% of patients stayed longer. This difference was attributed to the use of different cutoff points which were used to define the prolonged intensive care unit stay, which was 3 days.

This study revealed that the mortality rate of patients with prolonged ICU stays was 43.5% (95% CI = 31.9–55.1%), which means that nearly half of patients with prolonged stays died in the ICU. This finding was in line with those of studies conducted in Brazil^21^, Turkey^10^, Nigeria^24^, and South Africa^27^ which reported ranging from 31.1 to 44%.

This result was higher than that of studies conducted in America^30^, Switzerland^7^, Taiwan^31^, and Saudi Arabia^6^, which reported that mortality among patients with prolonged stays ranged from 4.7 to 21.1%. This result was lower than that of a study conducted in different hospitals in Turkey^3,12,18,20^ which revealed that the mortality of patients with prolonged stays ranged from 69.3 to 85.04%. This difference was attributed to the difference in the study population. In this study, the mean age of patients with prolonged stays was 42.14 ± 18.078 years. On the other hand, the mean age of the patients in those studies ranged from 64.3 ± 17.9 to 69 ± 22.5 years. This discrepancy may be due to the difference in life expectancy between the two countries. Those studies included more older participants than this study, which potentiated their mortality rates. The possible explanation for the older patients with prolonged stays having higher mortality was due to a weakened immune system, which predisposes them to a high risk for infections and other medical complications; the presence of comorbid illnesses; and the difficulty of recovering from the illness due to reduced functional status^33^.

After adjusting for potential confounders, sedatives/hypnotics, frequency of admission, and complications were found to be significantly associated with prolonged ICU stays at a P-value less than 0.05 in a multivariable binary logistic regression. The odds of staying longer in the ICU among those who took sedative agents were 2.75 times higher compared to those who did not take them. This finding is in line with several studies that reported that patients receiving sedation had a longer duration of mechanical ventilation and hospital stay in the ICU^34^. There are several possible explanations for sedative agents prolonging ICU stays. The pharmacokinetics of sedative agents can vary among patients based on their age, liver and kidney functions, and drug interactions; those factors affect metabolism and elimination, which prolong the effects of sedation^35^. In addition, oversedation can cause or worsen delirium, decrease respiratory drive, impair the cough reflex, delay weaning from mechanical ventilators, and reduce propulsive motility of the esophagus, which predisposes patients to micro aspiration of contaminated oropharyngeal secretions, which predisposes patients to ICU-acquired infections, which cumulatively increases the duration of stay on mechanical ventilation and in the ICU^34,36^.

The odds of staying longer among those readmitted to the ICU were 3.6 times higher than those who were first admitted to the ICU. This study result, in line with a study conducted at King Fahad National Guard Hospital in Saudi Arabia^6^, showed that readmissions was significantly associated with prolonged ICU stays. Other studies have shown that patients who were readmitted to the ICU had an increased length of stay in the ICU; for instance, a study was conducted in the United States of America^37,38^, Brazil^39^, and South Korea^40^. Patients are readmitted to the ICU for a variety of reasons, including experiencing complications during their initial stay or after discharge, disease progression, and premature or inappropriate discharge. These factors increase the likelihood of readmission. In addition to medical complications and their management, psychological impacts on patients who were readmitted can also play a significant role in prolonging their stay in the ICU.

Patients who developed complications stayed eight times longer than those who did not develop complications. This finding is consistent with a study conducted in the ICU of a third-level training and research hospital in Istanbul, Turkey^10^, which found that the development of acute kidney injury, pressure sores, and an increase in body mass index were associated with prolonged ICU stays. The possible explanations were that the management of complications requires more intensive care, aggressive treatment, and monitoring. In addition, having a longer recovery time contributes to a longer stay in the ICU. This result was supported by a study conducted by Iwashyna and Viglianti^41^, which revealed that the development of new complications obligates patients to stay longer.

The limitation of this study was that some laboratory tests were not recorded in the patients' charts. Despite these drawbacks, this study was the first multicenter investigation. Additionally, we included the most important factors related to an extended stay.

## Conclusion

According to the present study, one patient stayed longer than 14 days for every six patients admitted to the ICU, and nearly half of the patients who died during prolonged stays these patients need additional attention. The findings of this research boldly show the association of sedatives, readmissions to the ICU, and the occurrence of complications with the duration of ICU stays.

To reduce the length of stay in the ICU and improve the mortality rate, working toward those factors and optimizing ICU care are crucial. To determine the other factors related to the extended stay and early predisposing factors, further prospective studies with larger sample sizes are needed. In addition, to determine patients’ higher risk of prolonged stays in the ICU, studies need to be conducted using acute physiologic scores such as the SOFA and APACHE. Furthermore, investigating the effects of prolonged stays on quality of life and long-term outcomes crucial for patients, their families, and health professionals.

## Data Availability

The datasets created and analyzed during the current study are available from the corresponding author upon reasonable request.

## Abbreviations

AOR: Adjusted odd ratio
BSc: Bachelor of Science
CI: Confidence interval
GCS: Glasgow Coma Scale
ICU: Intensive care unit
MSC: Master of Science
WHO: World Health Organization
